# Quantum Crystallographic tomography of electrons in phase space

**DOI:** 10.1063/4.0000846

**Published:** 2025-10-27

**Authors:** M. Sizhuo Yu, Jean-Michel Gillet, Jules Andrevon-Canut

**Affiliations:** 1 CentraleSupelec, Gif sur Yvette, France, France; 2 Paris-Saclay University, Gif sur Yvette, France, France

## Abstract

Classical particles have been described in phase space since the early days of statistical physics. Each point corresponds to a given pair of position and momentum vectors. Describing electron behaviour in phase space is an obvious challenge to Heisenberg inequalities. Nevertheless, a full quantum description was proposed by E. Wigner in the early 1930s so that a function W(r,p) could thoroughly account for both the resulting position and momentum probability densities. It was shown to be helpful to compute the mean value of any observable (in pure or mixed quantum state). While the Wigner function has become popular in the quantum optics community, no phase-space experimental picture of electrons in a molecular crystal has ever been shown. A possible reason is that no single experimental technique gives access to electron phase-space representation. It is now apparent, just like any tomography, that only a combination of experimental data taken from different perspectives of phase space can yield a reliable representation of W(r,p) as the quasi-probability distribution. The challenges, methods and stakes of such a novel reconstruction will be illustrated, and our latest results (see figure) will be discussed.

